# Metastatic Mucinous Adenocarcinoma of Gastrointestinal Origin: A Rare Presentation of Peritoneal Carcinomatosis in a Woman

**DOI:** 10.7759/cureus.22517

**Published:** 2022-02-23

**Authors:** Krista M Wong, Mohammad Haidous, Zain AlShanableh, Poornanand Palaparty, Keyvan Ravakhah

**Affiliations:** 1 Internal Medicine, St. Vincent Charity Medical Center, Cleveland, USA; 2 Hematology/Oncology, St. Vincent Charity Medical Center, Cleveland, USA

**Keywords:** omental caking, peritoneal implants, appendiceal cancer, ovarian cancer, cancer of unknown primary, malignancy, peritoneal carcinomatosis, colorectal cancer, gastrointestinal cancer, metastatic mucinous adenocarcinoma

## Abstract

Peritoneal carcinomatosis is most commonly a rare late-stage manifestation of disseminated ovarian cancer. Women with peritoneal carcinomatosis with no obvious primary tumor are presumptively treated for ovarian cancer. However, less frequently, gastrointestinal cancer disseminates to the peritoneum, which would confer other treatment options. Herein, we present a case of a 68-year-old woman who was managed for peritoneal carcinomatosis of metastatic mucinous adenocarcinoma of lower gastrointestinal origin.

## Introduction

Peritoneal carcinomatosis is most commonly a rare late-stage manifestation of disseminated ovarian cancer, accounting for approximately 48% of cases [1]. Women with peritoneal carcinomatosis with no obvious primary tumor are presumptively treated for ovarian cancer. However, less frequently, gastrointestinal cancer disseminates to the peritoneum. 

The incidence of peritoneal carcinomatosis in colorectal cancer is between 5% to 8% but accounts for 2 to 3 out of 100,000 individuals per year because of the higher occurrence rate of colorectal cancer [2]. In 20% to 25% of patients with peritoneal carcinomatosis from colorectal cancer, the tumor is confined to the peritoneum in the absence of other metastasis [3]. However, the prognosis remains poor with a median survival of four to seven months without any treatment, but may be extended up to 12 months with palliative systemic therapy [4,5].

Herein, we present the case of a 68-year-old woman who was found to have peritoneal carcinomatosis and was initially presumed to have metastatic ovarian cancer. However, the pathological work-up eventually revealed metastatic mucinous adenocarcinoma of lower gastrointestinal origin, including appendix and colon. This case aims to bring awareness to the rare presentation of peritoneal carcinomatosis of gastrointestinal origin that is sparsely mentioned in literature and highlight the current, evolving treatments.

## Case presentation

A 68-year-old woman with a past medical history of type two diabetes mellitus, chronic obstructive pulmonary disease, irritable bowel syndrome, gastroesophageal reflux disease, and schizophrenia presented to the emergency department with a one-year history of intermittent, sharp lower abdominal pain. She further reported associated constipation, fatigue, loss of appetite, and a three-month history of unintentional 30-pound weight loss. She denied abdominal distension, bowel changes, fever, chills, nausea, vomiting, chest pain, shortness of breath, urinary changes, or postmenopausal vaginal bleeding. Negative past surgical history. Family history was significant for pancreatic cancer in mother, prostate cancer in father, gastric cancer in maternal aunt, and negative colorectal cancer history. She was an active smoker of ½ pack per day for the past 20 years. She denied illicit drug use or alcohol use.

The patient’s vital signs were stable. On physical exam, the abdomen was soft with mild lower abdominal tenderness upon deep palpation. A firm mass was palpated in the right lower quadrant. There was no abdominal distension or hepatosplenomegaly. Bowel sounds were present. Pelvic exam was unremarkable.

Laboratory results on admission were unremarkable. Tumor markers revealed mild elevations of carcinoembryonic antigen (CEA) 6.5 ng/mL (reference range: 0.0-4.7) and cancer antigen 19-9 (CA 19-9) 40 U/mL (reference range: 0-35). Alpha fetoprotein (AFP) and cancer antigen 125 (CA 125) were within normal limits.

**Figure 1:**
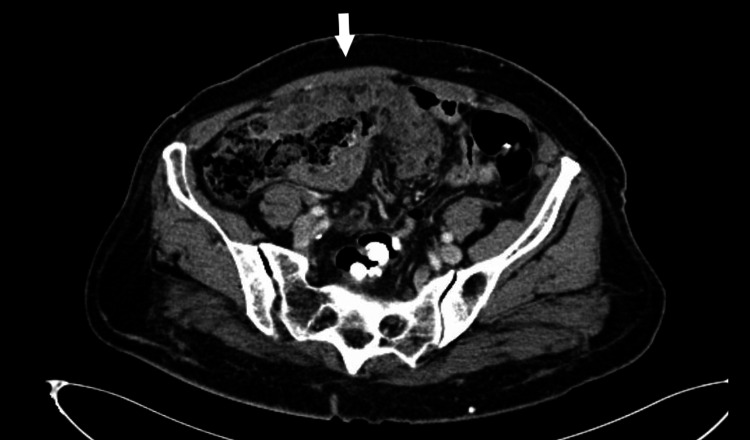
Contrast-enhanced computed tomography of the abdomen and pelvis Axial section demonstrating omental caking (white arrow).

**Figure 2:**
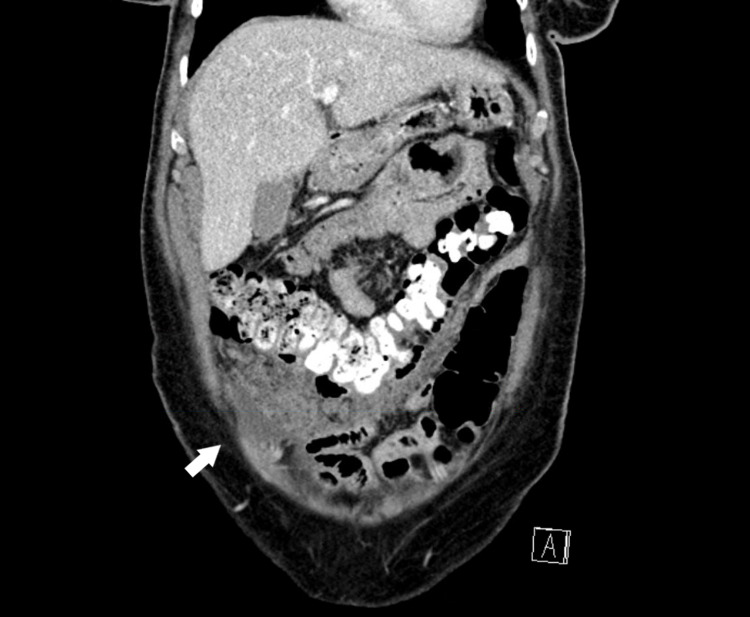
Contrast-enhanced computed tomography of the abdomen and pelvis Coronal section demonstrating omental caking concentrated in the right lower quadrant (white arrow).

Transvaginal ultrasound revealed no masses in the uterus or ovaries. CT scan of the abdomen and pelvis revealed peritoneal carcinomatosis most heavily concentrated in the right lower quadrant, small amount of ascites, and an enlarged right ovary (Figures 1-2). No lymphadenopathy was visualized. CT scan of the chest was unremarkable. Esophagogastroduodenoscopy (EGD) and colonoscopy were negative for malignancy.

**Figure 3:**
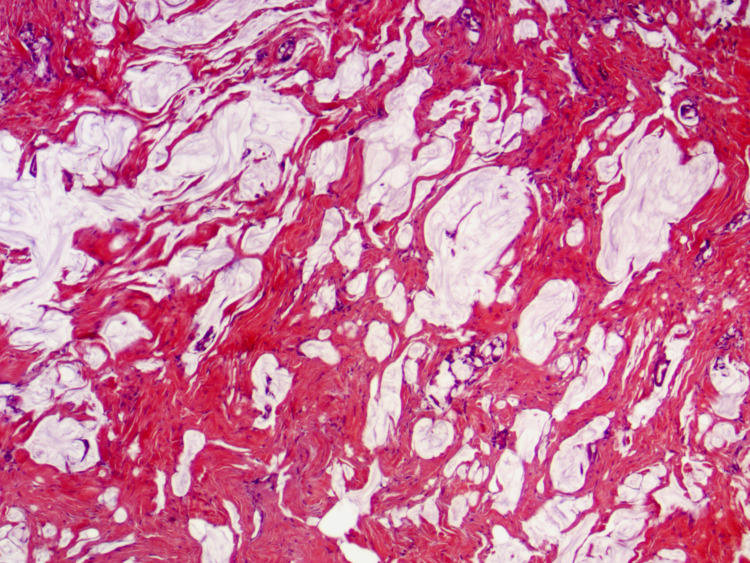
Peritoneal implants biopsy with hematoxylin and eosin (H&E) stain Peritoneal implants biopsy showing metastatic mucinous adenocarcinoma.

Diagnostic laparoscopy confirmed peritoneal implants, omental caking, and an enlarged right ovary. Biopsy of the peritoneal implants revealed metastatic mucinous adenocarcinoma (Figure 3). Biopsy of the right ovary was negative for malignancy. Immunohistochemistry studies showed positivity for cytokeratin 7 (CK7), cytokeratin 20 (CK20), caudal-type homeobox 2 (CDX2), and special AT-rich sequence-binding protein 2 (SATB2) with negative staining for paired-box gene 8 (PAX8) that was suggestive of lower gastrointestinal tract origin, including appendix and colon. The morphology and immunoprofile was not typical for mucinous tumor of ovarian origin. The patient was managed for peritoneal carcinomatosis of gastrointestinal origin. She was deemed to be a poor surgical candidate and was started on FOLFOX (folinic acid, 5-fluorouracil, oxaliplatin) chemotherapy.

## Discussion

Patients with peritoneal carcinomatosis present with nonspecific symptoms as the disease is often secondary to advanced ovarian cancer and less frequently, gastrointestinal cancer. Women with peritoneal carcinomatosis of unknown primary site are often treated for advanced ovarian cancer. However, it is important to distinguish between these cancers to guide appropriate management. Herein, we presented a case of a woman with peritoneal carcinomatosis who was found to have metastatic mucinous adenocarcinoma of lower gastrointestinal origin.

Mucinous adenocarcinoma of colorectal cancer accounts for 10% to 20% of colorectal cancer patients and is more common in women [6]. It is more commonly found in the proximal colon at advanced stages [6], as seen in our patient. A few studies showed a higher risk of synchronous peritoneal metastases from colorectal cancer in mucinous adenocarcinoma, right-sided colon cancer, advanced tumors, and younger patients [7-9]. 

Historically, peritoneal carcinomatosis has been considered a terminal disease. Treatment is generally with palliative systemic chemotherapy for advanced colorectal cancer. In the more recent years, there has been a shift towards considering localized peritoneal carcinomatosis with no distant metastasis as a locoregional disease [1,10-12]. The emerging strategy with curative intent for select patients is through the use of cytoreductive surgery (CRS) with hyperthermic intraperitoneal chemotherapy (HIPEC). Although some recent studies have suggested survival benefits in select patients with locoregional disease, there is no consensus on optimal selection factors for CRS and HIPEC.

## Conclusions

This case highlights the rare presentation of peritoneal carcinomatosis in a woman with metastatic mucinous adenocarcinoma of gastrointestinal origin. It is imperative for healthcare professionals to be aware of the other malignancies that can present as peritoneal carcinomatosis and the current treatment options to provide appropriate management for patients.
